# Cassava Starch as an Effective Texture Corrector of Fat-Free Dairy Products Based on Symbiotic Starter Culture

**DOI:** 10.1155/2022/1087043

**Published:** 2022-06-09

**Authors:** Maxim S. Tsyganov, Galina O. Ezhkova, Maya A. Kharitonova, Elena V. Nikitina

**Affiliations:** ^1^Kazan National Research Technological University, Kazan 420015, Russia; ^2^Institute of Fundamental Medicine and Biology, Kazan Federal University, Kazan 420008, Russia

## Abstract

Cassava starch can be an effective texture corrector for dairy products; however, the presence of a starchy taste in such products is undesirable. A possible solution to this problem can be the use of partially hydrolyzed cassava starch; complex microbial amylase preparations, for instance, Amylosubtilin® or *Bacillus licheniformis* amylases, can be used as an enzyme. The use of these enzyme preparations in low concentration allows it to obtain cassava starches with increased solubility, which can be easily used in the technology of dairy products. Starch is partially hydrolyzed by amylase preparations and does not significantly affect the chemical composition of the protein part of the Symbilact dairy product. Positive dynamics of the rheological and antioxidant properties of the low-fat dairy product “Symbilact” from the enzyme-modified cassava starch during storage were revealed in the researches. AT starches are more able to correct the structure, especially AT-0.5 and AT-1, but to a less extent increase antioxidant properties. As matter of a fact, BT starches exhibit higher antioxidant potential, but less structure correction.

## 1. Introduction

Russia and Europe produce commercial dairy products based on symbiotic and probiotic starter broth [1]. Such starter broths allow for expanding the existing range and market of dairy products. Microorganisms of the “traditional” yogurt starter culture *Lactobacillus delbrueckii ssp. bulgaricus*, *Lactobacillus acidophilus*, *Streptococcus thermophilus*, and *Bifidobacterium animalis* subsp. *lactis* are very useful: they affect commodity indicators and increase the utility and nutritional value of the product, as well as its safety. Species of the genus Lactobacillus contribute to the organoleptic properties of fermented foods and participate in the formation of texture [2]. Many lactic acid bacteria (LAB) secrete exopolysaccharides (EPS) [3]; they affect the textural, microstructural, and physical properties of yogurt, can increase water binding capacity and viscosity, and improve sensory characteristics, demonstrating excellent biocompatibility and the ability to stabilize milk emulsion [4]. *Lactobacillus delbrueckii ssp. bulgaricus* improves the structure of fermented milk products, increases stability, prevents syneresis [5], exhibits an immunostimulating effect [6], and is able to survive when passing through the gastrointestinal tract [7]. *Lactobacillus acidophilus* imparts a pleasant taste and aroma, increases stability, and is able to utilize fructooligosaccharides [8] and other prebiotic substances; in addition to exhibiting antidiabetic and antioxidant activities [9], they can be used in symbiotic functional products. *Streptococcus thermophiles* produces acetaldehyde, which in combination with milk thermolysis products results in the “typical” yogurt flavor. Some *Streptococcus thermophilus* strains produce exopolysaccharides [10]. *Bifidobacterium animalis* subsp. *lactis* is used as live cultures in fermented dairy products that serve as carrier products for them [11]. Bifidobacteria have a beneficial effect on the intestinal microbiota, have anticarcinogenic activity, improve the digestibility of lactose, reduce cholesterol levels [12], synthesize B vitamins, and facilitate calcium absorption [13].

One example of a symbiotic probiotic dairy product is the “Symbilact.” Its starter broth has a complex composition; they are the following: LAB *Lactococcus lactis ssp. cremoris*, *Lactococcus lactis ssp. lactis* var. *diacetylactis*, and *Lactococcus lactis ssp. lactis*; according to the literature, they have antagonistic activity to the pathogenic microbiota of the gastrointestinal tract [14]. Bacteria of the *Lactococcus* species improve the taste, aroma, and nutritional value of the fermented milk product [15]. *Lactococcus lactis* is used as a potential probiotic strain [16]*. Lactococcus lactis ssp. lactis* var. *diacetylactis* is capable of producing diacetyl from citrate, giving a nutty, creamy, and buttery aroma. *Lactococcus lactis* subsp. *lactis* can cause fruity aromas from esters, but it can produce bitter peptides from proteins. *Lactococcus lactis* subsp. *cremoris* breaks down bitter peptides, which prevents the appearance of a bitter taste in the product [17].

Thanks to this combination of microorganisms, Symbilact is recommended in Russia in baby and dietary nutrition; the product has a pleasant sour milk taste and thick consistency. Traditionally, the fat content of dairy products ranges from 2.5 to 4%.

Over a long period of time, nutritional standards and the relationship between diet and health have been established [18]. The dietary habits and lifestyle of consumers are changing: insufficient physical activity is revealed, the tendency to self-medication is growing, and the life expectancy of a person is increasing, which creates an additional burden on health care [19]. Diseases such as obesity, cardiovascular disease, chronic respiratory disease, diabetes, cancer, and liver and gastrointestinal tract diseases are the largest cause of death worldwide [20].

In this regard, it is important to release on the mass market useful symbiotic low-fat dairy products in order to meet consumer preferences and improve the health of the population. Fat in “traditional” fermented milk drinks, with a mass fraction of fat from 1.5 to 3.2%, plays the role of a thickener and structure-forming agent. The absence of fat globules leads to sensory and textural changes and, especially, a change in the perception of taste in the mouth [21], which is undesirable for the consumer [22].

In view of this, there is a need to improve the existing technology and the introduction of additional thickening agents in products with low fat content. The problem of “thickening” food is not new. Producers face it when producing yogurts with sweet fruit and berry fillings, where sugar has a “thinning” effect [23].

For natural thickeners and texture correctors [23], these are mainly bacterial biopolymers, hydrocolloids of animal and plant origin, and, in particular, native and modified starches from various botanical sources. Starch in the dairy industry is used to correct texture and extend shelf life [23].

The approach implemented in this study assumes the use of enzymatically modified starch in the technology for the production of fat-free “Symbilact.” Cassava (*Manihot esculenta Crantz*) is a perennial woody shrub with tuberous roots in the family Euphorbiaceae [24]. The plant name varies by region of cultivation: yucca (Central America), mandioca or manioca (Brazil), tapioca (India and Malaysia), and cassada or cassava (Africa and Southeast Asia) [25]. Cassava root contains up to 80% starch of dried weight [24]. The smooth surface of the granules (compared to potato starch); low residual amounts of lipids, proteins, and ash; lower amylose content (than other amylose-containing starches) and high molecular weight of amylose and amylopectin contained; and its mild taste make tapioca a unique natural starch for use in foods and industrial applications, as well as an excellent starting material for modification into special products [24, 25]. Tapioca starch is characterized by its gel-forming ability (may slowly form a weak gel); tapioca starch paste is still clearer, than those of cereal starches; setback or retrogradation is less noticeable in tapioca starch than is found in other starches [25]. Cassava (tapioca) starch in native or modified forms has nutritional benefits such as cholesterol-lowering, hypoglycemic, and antidiabetic effects [24]. These properties favor the use of tapioca starch over its cereal counterpart.

The purpose of this work is to study the properties of enzymatically modified cassava starch and study the effect of this starch on the technological (including rheological) and sensory characteristics of the nonfat sour milk drink “Symbilact.” According to the quality indicators of fat-free fermented milk products, identify modified starches that are most promising for use in nonfat dairy products from the point of view of a corrector of textural and organoleptic characteristics and protective properties.

## 2. Materials and Methods

### 2.1. Materials and Strains

The cow's skimmed UHT milk (fat 0.05%, Valio, Russia) was used for Symbilact. Milk totals are 0.16% fat, 3.18% protein, and 4.7% lactose. Cassava starches that were used in the experiment are the following: native GOST 32902-2014 (Origin Thailand, manufacturer in Russia LLC “Garnets”); enzyme-modified cassava starches AT-0.05, AT-0.1, AT-0.25, AT-0.5, and AT-1, obtained under the action of various concentrations of Amylosubtilin® (Berdsk Factory of Biological Preparations (now Sibbiofarm)), Russia; G3x (A -1500 U/g); and enzyme-modified cassava starches BT-0.05, BT-0, 1, BT-0.25, BT-0.5, and BT-1, obtained under the action of different concentrations of bacterial amylase *Bacillus licheniformis*. The *Bacillus licheniformis α*-amylase preparation was obtained in accordance with the method of Vafina et al. [26]. Commercial freeze-dried broth “Symbilact” (Ukraine, “VIVO Industry”) (strains *Streptococcus thermophilus*, *Lactobacillus delbrueckii* subsp. *bulgaricus*, *Lactobacillus acidophilus*, *Bifidobacterium animalis* subsp. *lactis*, *Lactococcus lactis subsp. cremoris*, *Lactococcus lactis subsp. lactis*, *Lactococcus lactis subsp. lactis* var. *diacetylactis*, and *lactose*) was used to produce dairy products.

### 2.2. Starch Modification

The starch modification was carried out as before [26] with modification. Native cassava starch was used for the experiment. Modification of this starch was based on the application of Amylosubtilin® (AM-starch) and amylase from *Bacillus licheniformis* (BT starch) of different concentrations (Table 1). Modification was carried out in water (30 g/100 ml), pH = 7, 40°C for 1 hour. The hydrolysis reaction was stopped with the addition of concentrated sulfuric acid (pH = 2). The starch was afterwards separated from the liquid by filtration and dried at 40°C. The starches were used for further research.

### 2.3. Analysis of Physicochemical Properties of Starches

Amylose content was determined by the iodine binding method described by Williams et al. [27] with modification. 20 mg of starch is mixed with 10 ml of 0.5 *n* KOH in a volumetric flask and mixed for 5 min, then brought to 100 ml with distilled water. From this volume, 10 ml is taken and mixed with 5 ml of 1N HCl and 0.5 ml of iodine solution (kJ -20 g and 2 g of J_2_ dissolved in 100 ml of distilled water) and brought up to 50 ml with distilled water and measured at 625 nm after 5 minutes. Calculation of the amylose percentage is calculated by the formula: *C*_A_ = 85.24 × −13.19.

The amount of protein was measured using the method described in Bradford [28]. Residual reducing sugar (residual dextrose) determination was analyzed using the 3,5-dinitrosalicylic acid (DNS) method [29]; DNS reacts with glucose to form a colored complex. The color intensities were measured by a spectrophotometer at 575 nm.

The methods described by Omojola et al. [30] were used to determine the water absorption capacity (WAC) and water solubility index (WSI). For WAC, the starch sample (5% *w*/*v*) was dispersed in a preweighed centrifuge tube. The tube was placed in a vortex mixer for 2 min, left at room temperature for 30 minutes, gently stirred during this period, and then centrifuged at 3000 rpm for 15 minutes. The supernatant was then discarded, and the weight of the tube and hydrated sample was measured. The weight was calculated and expressed as the weight of water bound by 100 g dry starch.

WSI was the weight of dry solids in the supernatant from the WAC test. The supernatant liquid was then decanted into a tared evaporating dish of known weight. The weight of dried solid after evaporation in an air-circulating oven at 103°C was recorded. The WSI (expressed as grams solids/100 g of starch) was calculated from the weight of dry solids recovered by evaporating the supernatant at 110°C for 12 h.

Gelatinization temperature was evaluated using the method of Attama et al. [31]. The starch sample (1 g) was put in a 20 ml beaker, and 10 ml of distilled water was added. The dispersion was heated on a hot plate. The gelatinization temperature was then measured with a thermometer suspended in the starch slurry. Dynamic viscosity was evaluated for the 1% starch solution, pregelatinized, and cooled down to 20° C, using the VNZh (Russia) glass capillary viscosity meter, tracking the starch gel flow time.

### 2.4. Obtaining a Thermostatic Product

Starches (native, АT-0.05, АT-0.1, АT-0.25, АT-0.5, АT-1, ВT-0.05, ВT-0.1, ВT-0.25, ВT-0.5, and ВT-1) were added to cow's skimmed milk (fat 0.05%, Valio, Russia) at a concentration of 1% and subjected to heating, in a boiling water bath followed by heating at 100°C for 30 min; the mixture was stirred for uniform distribution of starch. The lyophilized dried broth for Symbilact was added to nonfat milk at the rate of 0.1 g/100 ml and fermented at 37°C for 16 hours. Then, it was added as a broth to milk mixtures with starches at the rate of 5% by weight of the mixture. To obtain a fermented dairy food, milk was fermented at 40°C for 8 hours; then, the finished product was cooled at 4–5°C for 20 h for stabilization. Then, the samples were analyzed (1 day), the batch of yogurt was left for storage at 4°C for 28 days, and measurements were also carried out after storage.

### 2.5. Physicochemical Analyses of Symbilact

Analysis of proteins, dry matter, and density in the Symbilact has been described by us earlier [32]. Salts, proteins, and carbohydrates in whey were tested in whey of the Symbilact after centrifugation at ambient temperature at 3000 g for 15 min by milk analyzer “Klever-2M” (Russia). Acidity analysis (pH measurements and the titratable acidity) of Symbilact also has been described by us earlier [32]. Titratable acidity of Symbilact samples was expressed in Thörner degrees.

### 2.6. Texture Parameters

For water holding capacity (WHC) and syneresis analysis [30], samples of Symbilact (about 20 g) (*Y*) after cooling to +4°C in 24 hours of storage were centrifuged for 10 minutes at 3000 rpm, +20°C. The released whey (*W*) was removed and weighed. The WHC of fermented milk is calculated as follows:

(1) $$\begin{array}{c} \text{WHC}=\frac{Y-W}{Y}100\%. \end{array}$$

Syneresis was measured after samples (about 20 g) were cooled to +4°C in 24 hours of storage (*Y*). Samples were centrifuged for 5 minutes, 500 rpm in +20°C. The released whey (*S*) was removed and weighed. Syneresis of fermented milk is calculated as follows:

(2) $$\begin{array}{c} \text{Syneresis}=\frac{S}{Y}100\%. \end{array}$$

For apparent viscosity of fermented milk, the apparent viscosities of the Symbilact samples (100 ml) were measured at 8°C using a Brookfield concentric cylinder viscometer (model RV-DVIII, Inc., China) equipped with rotor no. 3, mixing at 60 rpm. Measurements were carried out in triplicates for each treatment, and results were recorded in mPa·s.

### 2.7. Sensory Evaluation

Symbilact samples were evaluated as before by the panelist [32]. At least 10 participants in three replicates evaluated samples. The color parameters (*L*∗, *a*∗, and *b*∗) of the Symbilact samples were measured using a colorimeter Chroma Meter (China) [33]. Different values represent different colors: *L*∗, darkness-lightness (0–100); *a*∗, greenness-redness (−60–+60); and *b*∗, blueness-yellowness (−60–+60). Symbilact samples (50 ml) were stirred and placed in an aluminium cylinder (outside diameter 55 mm), with the surface optically flat before measuring, and the sensor was mounted directly on top of the cylinder to prevent ambient light noise.

### 2.8. Evaluation of Radical-Scavenging Ability (RSA) by 2,2-Diphenyl-1-picryl Hydrazyl (DPPH) Assay

Antioxidant scavenging capacity was analyzed according to the procedure described by [34], with some modifications. Samples of Symbilact were previously diluted 10 times, and their whey was diluted 5 times, after which 1 ml diluted samples were mixed with 1 ml of freshly prepared DPPH solution (0.12 mM) in ethanol. The reaction mixtures and the control (2 ml; DPPH in ethanol) were incubated at room temperature in the dark for 30 min. Mixtures are centrifuged at ambient temperature for 2 minutes, 10000 rpm. The absorbance of the supernatant was measured at max 517 nm using a spectrophotometer. The DPPH radical scavenging activity was calculated with the following equation: DPPH∗scavenging activity% = [(control absorbance − extract absorbance)/(control absorbance)] × 100%.

### 2.9. Ferric Reducing Antioxidant Power (FRAP) Assay

The FRAP assay was carried out following the procedure described by [35] with modifications. 1 ml of the test product (whey) was mixed with 1 ml of 0.2 M potassium sodium phosphate buffer (pH 6.5) and 1 ml of 1% potassium ferricyanide. The reaction mixture was incubated for 20 min at 50°C and cooled, after which 1 ml of 10% trichloroacetic acid was added. The mixture was centrifuged at 2000 rpm for 10 min at room temperature. The supernatant (2 ml) was added 2 ml of distilled water and 400 *μ*l of 0.1% FeCl_3_. The control was prepared similarly; only 1% potassium ferricyanide was replaced with a buffer mixture. The absorption of the reaction mixture was measured at 700 nm. The reducing force was expressed as absorbance at 700 nm relative to the control.

### 2.10. Statistical Analysis

All experiments were carried out in triplicate. Five replicates were performed for DPPH analysis. Significance was established at *P* < 0.05. The results were analyzed for statistical significance with two-way ANOVA by GraphPad Prism software at a significance level of *P* < 0.05. The graphical representation of the principal component analysis (PCA) allows the data to be analyzed on a two-dimensional P1/P2 map by Statistica12 (Statsoft) and to identify the trends between variables at a significance level *P* < 0.05. When two variables are far from the centre and close together, they correlate significantly. If they are on the opposite side of the centre, then they are significantly negatively correlated.

## 3. Results

### 3.1. Properties of Fermented Modified Cassava Starches

Physical-chemical parameters and functional properties of native and fermented modified cassava starches are presented in Table 2. The percentage of amylose in cassava starches modified by *Amylosubtilin*® and *Bacillus licheniformis* amylase increased by an average of 4 times in comparison with the native one. The highest percentage of amylose was in starches treated with an average concentration of the enzyme (AT-0.25, BT-0.1). An increase in the concentration of *Bacillus licheniformis* amylase above 0.830 U/g starch led to a decrease in the proportion of amylose and, apparently, due to its degradation under the action of the enzyme. The content of amylopectin in the samples, respectively, was inversely proportional to amylose. The amount of residual free dextrose in modified starches decreased (1.73-6.27 g dextrose/100 g starch) compared to native (10.55 g dextrose/100 g starch). Its minimum amount was in samples AT-1, BT-1, and BT-0.05 (Table 2).

The dynamic viscosity of the modified starches was lower than that of the native one. The lowest viscosity was found in starch samples treated with average enzyme concentrations (AT-0.25, BT-0.1, and BT-0.25).

The enzymes used to modify the starch had different effects on the gelatinization temperature. An increase of Amylosubtilin® concentration during modification led to a monotonic decrease in the temperature of gelatinization of polysaccharides. In the case of BT starches, an increase in the enzyme concentration led to the opposite effect.

The WAC of starches after modification increased: 2.88-4.88 g H_2_O/g starch in the modified versus 2.4 g H_2_O/g starch in the native. The largest increase in WAC was observed for AT-0.5 samples, BT-0.5, and BT-1. The WSI value of the modified starches was higher than that of the native starches. High concentrations of the enzyme amylase *Bacillus licheniformis* (2.07 U/g starch and higher) lead to greater degradation of starch, converting them into a more soluble form. Thus, the action of enzymes on starches caused an increase in WAC and WSI.

### 3.2. Physicochemical Properties of the Dairy Products

At the next stage of the study, starches were used to correct the texture of the nonfat sour milk drink Symbilact.  Table 3 indicates the changes in pH and titratable acidity of Symbilact samples at storage times. The pH of Symbilact-native was 4.72, which is higher than in other samples on day 1. After 28 days of storage, the pH of all samples decreased due to postacidification processes due to the residual activity of microorganisms even at low storage temperatures. The titratable acidity was inversely proportional to the pH value. The acidity of control and Symbilact-native increased during storage (Table 3). On the contrary, the acidity of Symbilact-AT decreased for all samples. In the Symbilaсt-BT series, both an increase (BT-0.25; BT-0.5) and a decrease in titratable acidity (BT-0.1; BT-1) were observed. The titratable acidity of the Symbilact sample BT-0.05 remained unchanged, which indicates the stability of the sample during storage. The decreased pH of Symbilact during refrigerated storage is caused by the growth and activity of starter bacteria which remain active during storage.

The physicochemical composition of Symbilact with different starches is shown in Table 4. The addition of starches led to an increase in the density of the product, which changed little during storage. The content of solid matter, salts, and density, as expected, increased in the samples with starches, which was a consequence of their introduction in the amount of 1% to the total mass.

The amount of protein in the product with modified starches was slightly higher than that in the control and Symbilact-native, which is possibly related to an increase in the density of the milk curd. The higher total protein, dry matter, and salt content are due to the increased density of Symbylact due to the addition of starch stabilizers. Probably, starch changing water activity influences redistribution of milk protein fractions. On the 28^th^ day of storage, the total protein in Symbilact-AT and BT was in the range of 3.94-4.16%. The added modified starches resulted in an increase in the proportion of proteins in whey. Probably, starch changing water activity influences redistribution of milk protein fractions. After 28 days of storage, the content of this protein decreased in all samples but remained still higher in the samples with modified starches compared to the control. As indicated, the Lactococcus cell includes many proteolytic enzymes that can hydrolyze caseins and peptides.

Perhaps, the presence of modified starches and their incorporation into the matrix of the milk gel contribute to the viability of LAB; as a result, autolytic processes are inhibited and the intracellular enzyme output is suppressed. There is protein conservation in samples with modified starches.

In the control, the carbohydrate content is the lowest. Naturally, the introduction of starches as a carbohydrate component increased the level of carbohydrates; in Symbilact-AT and BT, this indicator was in the range of 5.12-5.46%. The largest amount of carbohydrates is in Symbilact-native, which is probably due to the fact that the introduced starch was the least water-soluble. During fermentation and storage, modified starch is more easily hydrolyzed by LAB than native starch, so modified starch can act as an additional carbohydrate substrate, resulting in lactic acid synthesis being intensified. In fact, after 28 days of storage, the amount of carbohydrates in all samples with modified starches decreased. In Symbilact-native, after 28 days, there was the highest value, which may be due to the inaccessibility of native starch for destruction by lactic acid bacteria.

### 3.3. Textural Properties of the Dairy Products

**Figure 1:**
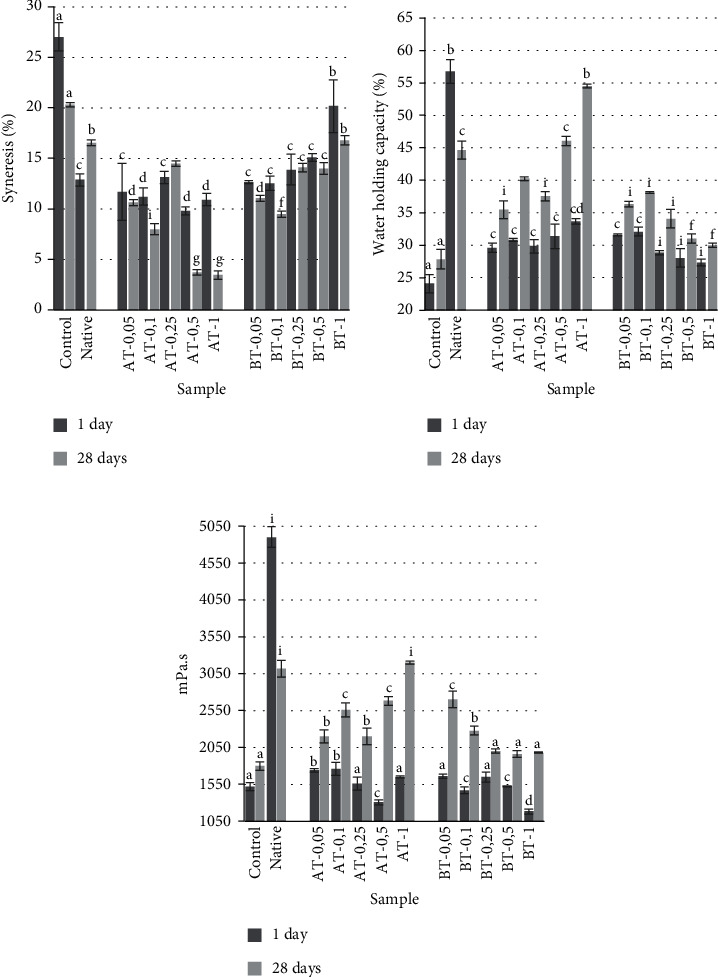
Syneresis (a) and WHC (b) and apparent viscosity (c) of Symbilact samples with added different starches. Data are the mean ± standard deviation of three independent replicates (*N* = 3). Different letters in the same column (after 1 or 28 days) indicate significant differences (*P* < 0.05).

The apparent viscosity of the fat-free dairy product is low as there are no fat globules. Lack of fat provokes an increase in syneresis. The addition of enzymatically modified cassava starches had a positive effect on the level of syneresis in the samples (Figure 1(a)). On the first day of storage, the syneresis of Symbilact with starches was in the range of 9.8-20.2%; in the control, 27%. After 28 days of storage, syneresis decreased in all samples, except for Symbilact-native, where this indicator increased. The AT-1 sample had the minimum syneresis at the end of the shelf life.

WHC of Symbilact was inversely correlated with syneresis (Figure 1(b)). The smallest WHC after 1 day of storage was found in Symbilact-control—24.1%; the highest is in Symbilact with native cassava starch—56.7%, which is associated with the high swelling capacity of whole starch granules. WHC of Symbilact-AT and BT was in the range of 27.3–33.7%, which is higher than the control. After 28 days of storage, the WHC of all samples increased, with the exception of Symbilact-Nat, in which the WHC decreased as a consequence of the retrograde of native starch in the milk gel system; in addition, large granules of native starch are not capable of close interaction and the formation of strong bonds with the protein-milk gel. The highest WHC values were in the AT-0.5 and AT-1 samples. A similar trend to WHC was found for apparent viscosity (Figure 1(c)).

The introduction of native starch into Symbilact resulted in a 3.2-fold increase in viscosity in comparison with the control. The viscosity of the AT and BT samples was in the range of 1184-1760 mPa·s. After 28 days of storage, the viscosity of all samples with modified starches increased by 1.3-1.7 times; in the control, 1.2 times. In Symbilact with native starch, the apparent viscosity decreased by 1.5 times, probably as a result of starch retrogradation. Statistical analysis of the correlation index between WHC and apparent viscosity was 0.909.

Increasing the amount of solids by adding starches led to increased product viscosity; increased amount of lactic acid in variants with modified starches intensified destabilization of casein (paracasein) micelles to aggregate to form a gel. Partially hydrolyzed modified starch can more successfully fill the space between casein micelles than native starch.

### 3.4. Sensory Properties and Color of the Dairy Products

Samples of Symbilact with modified starches did not differ from the control in appearance (Table 5); they had a pleasant, pronounced fermented milk taste and aroma without foreign odors, bitterness, and aftertaste. The respondents revealed a slight sweetness in the samples—Symbilact AT-0.1, AT-0.5, AT-1, BT-0.1, BT-0.5, and BT-1. Symbilact-control and Symbilact with modified starches, in general, had a fine-grained, delicate, smooth, and creamy viscous texture without lumps. Symbilact AT-1 and BT-1 created the impression of the most delicate texture.

Symbilact with native cassava starch was the least preferable in appearance: it had a loose, nonuniform solid-jelly structure, exfoliated upon stirring, and created an unpleasant feeling of coarse fibers (clumpy texture) when chewed in the mouth. The Symbilact-Nat sample had a fermented milk aroma and an unexpressed taste with a starchy aftertaste.

The average taste rating of the Symbilact samples with FMS (FMC) was higher than the Symbilact control, due to their rich creamy taste. The highest total score (18-20 points) was received by the following samples: AT-0.1, AT-0.5, AT-1, BT-0.5, and BT-1. In the presence of modified starches, L of some samples of Symbilact (AT-0.05, A-0.1, B-0.1, and B-0.25) decreased. The shift in the color range of Symbilact-AT and BT to the red and blue sides may probably be associated with the Maillard reaction (the interaction of milk proteins with starch breakdown products). The addition of starches led to a decrease in the gloss of Symbilact.

### 3.5. Antioxidant Properties of the Dairy Products

**Figure 2:**
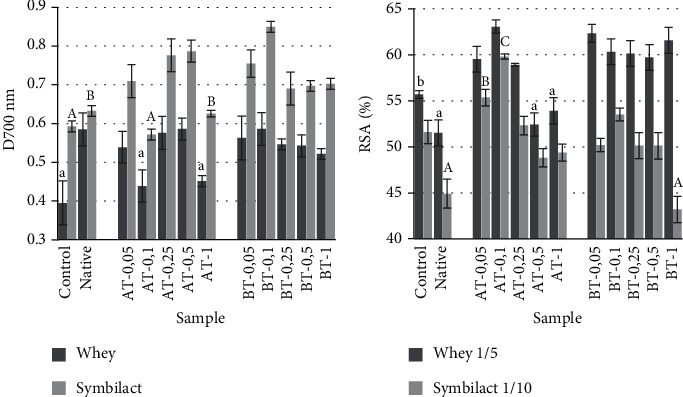
FRAP (a) and DPPH-scavenging activity (b) of *Symbilact* samples added with different starches after 28 days of storage. Data are the mean ± standard deviation of independent replicates (*N* = 3 for FRAP, *N* = 5 for RSA). Different letters indicate significant differences (*P* < 0.05).

FRAP whey and product of Symbilact with starches at 28 days of storage were higher than that of the control (Figure 2(a)). The greatest reducing potential was shown by Symbilact with starches modified by *Bacillus licheniformis* amylase. The maximum FRAP of the product was found in BT-0.1 and in whey—in AT-0.5 and BT-0.1.

The addition of enzymatically modified starches led to an increase in whey RSA in most cases by 5-10% compared to control (Figure 2(b)). The use of native starch reduced the ability to bind free radicals in the whey and the product itself. Products with FMS in most cases showed RSA at the control level; however, the RSA of samples AT-0.05 and AT-0.1 was statistically higher.

### 3.6. Principal Component Analysis (PCA)

**Figure 3:**
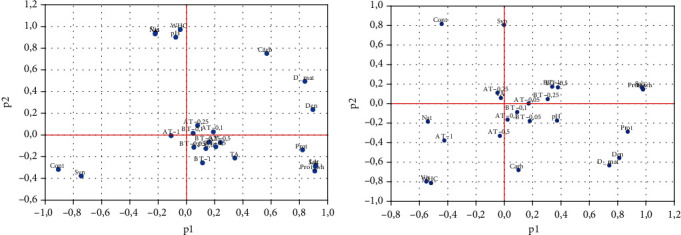
Principal component analysis (PCA) of Symbilact samples added with different starches after 1 (a) and 28 (b) days of storage. Cont, Nat, АT-0.05, АT-0.1, АT-0.25, АT-0.5, АT-1, BT-0.05, BT-0.1, BT-0.25, BT-0.5, and BT-1 are Symbilact samples. Prot: protein; Prot_wh: protein in whey; Carb: carbohydrates; Lac: lactose; salt: salts; D_mat: dry matter; Den: density; TA: titratable acid, pH; Syn: syneresis, WHC; Vis: viscosity.

PCA (Figure 3) showed that at 1 day of storage, the product Symbilact with modified starches (product indicators) AM and BT is in the same area, as close as possible to each other, indicating little change between them. Thus, Amylosubtilin or amylase *Bacillus licheniformis* concentrations for partial hydrolysis of starch during modification do not affect the quality of the fresh dairy products. After 28 days of storage, the distance between the samples with AM and BT samples increased, which indicates a change in the properties of the products and an increase in differences in the totality of the analyzed properties. The difference in the stabilizer used and its influence on the final properties of the product are revealed only after some storage time.

Indicators (in pairs) Vis and WHC, density and dry matter, and salts and proteins, between the vectors of which the angle is minimal, demonstrate a high degree of correlation between themselves both on days 1 and 28 of storage. The location of samples AT-0.05 and BT-0.05 changed minimally, which indicates the stability of the fermented milk product.

The syneresis (Syn) and carbohydrates (Carbon) indicators are negatively correlated, the angle between them is almost equal to 180°. The longest vector is more important (the indicator is farther from the centre of the graph). Thus, the analyses show that the most important are texture indicators (syneresis, viscosity, and WHC) and chemical composition (proteins, density, and dry matter).

## 4. Discussion

A number of researchers have recommended the use of cassava starch because of its neutral taste, high purity, viscosity, good solubility, and swelling capacity [36, 37]. The use of maltodextrin from cassava starch in the low fat yogurt technology resulted in a texture correction that was creamy smooth and rich in flavor [38].

In previous works, the results of the modification of potato starches with complex preparations of Amylosubtilin and *B. licheniformis* amylase, possessing high alpha-amylase activity, have been described [26]. The differences in AT and BT of cassava starches are explained by the presence of minor beta-amylase, cellulase, protease, and other activities in the used enzyme preparations (Table 2). Enzymatically modified cassava starches had different amylose/amylopectin ratios, which influenced their technological properties. The high WAC and WSI allowed us to assume that the use of such starches in the technology of fermented milk drinks is promising. Enzymatically modified starches did not inhibit the development of lactic acid bacteria; a higher pH and low titratable acidity were revealed in comparison with the control, while native starch reduced the rate of accumulation of lactic acid (Table 3). Such effect is associated with a decrease in water activity and blocking of the metabolism of lactic acid bacteria, which was previously reported by He et al. [39]. Postenzymatic processes during storage in the product are due to the residual activity of microorganisms, as evidenced by a decrease in pH and the amount of carbohydrates [40]. In addition, the postacidification activity can be due to the use of starches by bacteria as an additional source of energy (Table 2) [41]. The enzymatically modified starches can play the role of stabilizer in yogurt and can keep constant titrable acidity and buffering capacity during storage [42].

The addition of starch affects the redistribution of milk protein fractions towards an increase of protein in whey [43]. Starches change the activity of water and increase the local concentration of milk protein (casein micelles) during swelling due to the absorption of water from the continuous phase [44]. In addition, the development of lactic acid bacteria contributes to an increase in proteolytic activity, resulting in an increase in the number of peptides as a product of casein hydrolysis [41].

The improvement in texture of products with enzymatically modified starches is due to their low viscosity, increased WAC, and solubility (WSI) (Table 2). During enzymatic hydrolysis, morphological rearrangements occur, loosening starch granules [26], which increases the total contact area with dairy components in the dairy product. The addition of modified starches leads to a decrease in syneresis (Figure 1(a)) due to the ability of these starches to form complexes with proteins [45] and their increased swelling and water absorption [46].

The introduction of native starch, which has a high gelling ability and water absorption after heating, causes a high WHC level of Symbilact-Nat, but the retrograde starch during storage of native starch leads to texture defects. When using modified starches, the WHC of Symbilact increased, which may also be associated with the release of hydrophilic free amino acids and short-chain polypeptides during hydrolysis of whey protein [47] and with the formation of an interpenetrating continuous network between whey proteins and more open hydrolyzed starch granules [48].

An increase in the viscosity of Symbilact with modified starches was due to the ability of starch to swell [46], strengthening of the protein network due to the destruction of starch, solubilisation of amylose, and filling of the gel network [49]. In addition, the viscosity increases due to an increase in the total amount of polysaccharides [41], which can be located in the pores of the protein network [49] and are responsible for texture [50]. Earlier, Ibrahim and Khalifa [43] using microstructure analysis revealed that stabilizers including modified starches occupied an empty space in the network of casein particles; this interaction contributes to a decrease in serum separation in addition to the effect of increasing viscosity.

The textural changes due to the introduction of modified starches had a positive effect on the taste of Symbilact (Table 5). The respondents noted their creamy, smooth, and pleasant texture. This was in line with previous results for milk-based desserts [51], where a thicker, denser texture felt higher satiety and was also highly rated by the panelists.

The increase in the ability of the whey of Symbilates to bind free radicals may be due to antioxidant peptides secreted by lactic acid bacteria during milk fermentation [52] and exopolysaccharides (EPS), where the added starch promotes the development of starter cultures and the formation of a complex of these substances [41].

## 5. Conclusions

This study demonstrated changes in the chemical, rheological, and antioxidant properties of the nonfat dairy product Symbilaсt with the addition of enzymatically modified cassava starch. Enzymatically modified starches, unlike native starches, do not inhibit the acid-forming activity of lactic acid bacteria. Starches modified with Amylosubtilin® are better able to correct the structure, especially AT-0.5 and AT-1, but to a lesser extent increase the antioxidant properties of Symbilact. In contrast, BT (modified by amylase *Bacillus licheniformis*) starches exhibit higher antioxidant potential, but less structure correction. To sum up, the use of enzymatically modified cassava starches makes it possible to obtain a dairy drink Symbilact based on skim milk with improved texture properties, better taste (the consistency, aroma, and taste of the products were more attractive), and also high antioxidant potential.

## Figures and Tables

**Table 1:** Code of fermented modified starches.

Starches	Amylase activity (U/g starch)	Amylosubtilin (g/100 ml reaction mixture)	Amylase *Bacillus licheniformis* (ml/100 ml reaction mixture)
AT-0.05	0.415	0.0100	—
AT-0.1	0.830	0.0201	—
AT-0.25	2.070	0.0500	—
AT-0.5	4.150	0.1005	
AT-1	8.300	0.2010	—
BT-0.05	0.415	—	0.05
BT-0.1	0.830	—	0.1
BT-0.25	2.070		0.25
BT-0.5	4.150	—	0.5
BT-1	8.300	—	1

**Table 2:** Physical-chemical parameters and functional properties of native and fermented modified cassava starches.

Starch samples	Amylose (%)	Amylopectin (%)	Residual dextrose (g dextrose/100 g starch)	Dynamic (ml, sec)	Gelatinization temperature (°C)	WAC (g H_2_O/g starch)	WSI (g solids/100 g starch)
Native	5.20 ± 0.42^a^	94.69 ± 0.28^a^	10.55 ± 0.07^a^	8.43 ± 1.41^a^	52.9 ± 0.8^a^	2.40 ± 0.28^a^	4.72 ± 0.31^a^
АT-0.05	20.22 ± 2.55	79.37 ± 1.98	4.26 ± 0.23	2.74 ± 1.13	55.1 ± 1.4	3.57 ± 0.24	8.18 ± 0.25
АT-0.1	22.69 ± 2.84	76.90 ± 2.28	4.77 ± 0.03	3.17 ± 0.71	52.1 ± 1.9	3.21 ± 0.16	7.84 ± 0.28
АT-0.25	24.99 ± 0.72	74.45 ± 0.37	5.49 ± 0.04	2.03 ± 0.66^c^	52.2 ± 0.7	2.88 ± 0.11	5.93 ± 0.24
АT-0.5	23.54 ± 2.18	76.05 ± 1.61	4.26 ± 0.28	4.52 ± 1.27	51.8 ± 0.8	4.36 ± 0.08	7.81 ± 0.30
АT-1	22.26 ± 2.74	77.33 ± 2.18	1.73 ± 0.03	2.85 ± 0.99	50.9 ± 1.3	3.42 ± 0.06	7.65 ± 0.21
BT-0.05	22.69 ± 0.71	76.90 ± 0.14	3.89 ± 0.01^b^	3.08 ± 0.85	55.0 ± 1.4	3.60 ± 0.14	7.06 ± 0.23
BT-0.1	25.33 ± 0.47	74.66 ± 0.47	6.27 ± 0.06	1.44 ± 0.74^c^	54.8 ± 0.6	3.11 ± 0.18	7.08 ± 0.25
BT-0.25	23.71 ± 0.71	75.88 ± 1.27	5.10 ± 0.14	2.40 ± 0.78	55.8 ± 0.8	2.92 ± 0.25	23.72 ± 0.29^b^
BT-0.5	20.47 ± 0.28	79.42 ± 0.42^b^	5.31 ± 0.21	3.98 ± 0.99^b^	57.5 ± 1.0	4.59 ± 0.14	36.70 ± 0.21^b^
BT-1	16.47 ± 0.66^b^	83.42 ± 0.52^b^	3.97 ± 0.05^b^	4.33 ± 1.17	59.8 ± 1.2^b^	4.88 ± 0.10	49.34 ± 0.20^b^

Data are the mean ± standard deviation of three independent replicates (*N* = 3). Values in the same column having different letters differ significantly (*P* < 0.05).

**Table 3:** pH measurements and titratable acidity of Symbilact sample with added different cassava starches.

Symbilact sample	рН		Titratable acid (°Т)	
	1 day	28 days	1 day	28 days
Control	4.44 ± 0.03	4.25 ± 0.03	100.9 ± 0.1^a^	105.9 ± 0.6
Native	4.72 ± 0.01^a^	4.27 ± 0.01	100.7 ± 0.2^a^	102.3 ± 0.4^a^
АT-0.05	4.44 ± 0.00	4.24 ± 0.03	111.9 ± 0.1	108.0 ± 0.7
АT-0.1	4.49 ± 0.03	4.25 ± 0.00	113.8 ± 0.4	101.8 ± 0.4
АT-0.25	4.53 ± 0.01^b^	4.28 ± 0.03	111.8 ± 0.4	104.0 ± 0.0
АT-0.5	4.43 ± 0.03	4.28 ± 0.04	112.8 ± 0.4	107.9 ± 0.6^b^
АT-1	4.48 ± 0.03	4.24 ± 0.01	118.0 ± 1.4^b^	108.0 ± 0.0^b^
BT-0.05	4.40 ± 0.01	4.37 ± 0.01	100.9 ± 0.1^a^	101.0 ± 0.7^a^
BT-0.1	4.54 ± 0.01^b^	4.32 ± 0.00	111.8 ± 0.4	102.8 ± 0.4^a^
BT-0.25	4.52 ± 0.03	4.28 ± 0.06	101.0 ± 0.1^a^	107.0 ± 0.7
BT-0.5	4.52 ± 0.04	4.27 ± 0.04	100.8 ± 0.3^a^	105.0 ± 0.7
BT-1	4.42 ± 0.00	4.29 ± 0.01	116.8 ± 0.4^b^	104.7 ± 0.2

Data are the mean ± standard deviation of three independent replicates (*N* = 3). Values in the same column having different letters differ significantly (*P* < 0.05).

**Table 4:** Physicochemical composition of *Symbilact* sample with added different starches.

Symbilact sample	Proteins (%)		Proteins in whey (%)		Carbohydrates (%)		Salts (%)		Dry matter (%)		Density (kg/m^3^)	
	1 day	28 days	1 day	28 days	1 day	28 days	1 day	28 days	1 day	28 days	1 day	28 days
Control	3.81 ± 0.28	3.78 ± 0.31	2.77 ± 0.04^a^	2.61 ± 0.03^a^	4.67 ± 0.03^a^	4.64 ± 0.06^a^	0.65 ± 0.01^a^	0.66 ± 0.01^a^	9.52 ± 0.33	9.53 ± 0.28^a^	1032.3 ± 0.8^a^	1032.9 ± 0.8^a^
Native	3.87 ± 0.25	3.67 ± 0.28	2.88 ± 0.08^a^	2.71 ± 0.06^a^	5.64 ± 0.06^b^	5.45 ± 0.07^b^	0.68 ± 0.01^b^	0.63 ± 0.01	10.84 ± 0.20	10.35 ± 0.35	1037.5 ± 0.7	1035.1 ± 0.8
АT-0.05	4.05 ± 0.23	4.12 ± 0.30	3.12 ± 0.03	3.05 ± 0.07	5.12 ± 0.06	5.12 ± 0.03	0.73 ± 0.01	0.71 ± 0.01	10.50 ± 0.32	10.65 ± 0.36	1038.1 ± 0.7	1037.5 ± 0.7
АT-0.1	4.10 ± 0.28	4.13 ± 0.31	3.09 ± 0.06	2.95 ± 0.07	5.39 ± 0.03	5.08 ± 0.08	0.72 ± 0.01	0.69 ± 0.01	10.75 ± 0.23	10.55 ± 0.35	1037.8 ± 1.1	1037.6 ± 0.8
АT-0.25	4.03 ± 0.25	4.01 ± 0.22	3.04 ± 0.06	2.93 ± 0.04	5.46 ± 0.04	5.02 ± 0.03	0.71 ± 0.01	0.68 ± 0.01	10.84 ± 0.28	10.36 ± 0.36	1037.2 ± 0.9	1036.8 ± 0.8
АT-0.5	4.07 ± 0.30	4.15 ± 0.21	3.15 ± 0.07	2.91 ± 0.03	5.36 ± 0.08	5.08 ± 0.10	0.73 ± 0.00	0.68 ± 0.01	10.82 ± 0.35	10.56 ± 0.23	1037.2 ± 0.9	1037.7 ± 1.0
АT-1	3.86 ± 0.23	3.94 ± 0.28	3.02 ± 0.03	2.77 ± 0.04	5.12 ± 0.07	5.04 ± 0.06	0.70 ± 0.01	0.65 ± 0.01	10.35 ± 0.28	10.28 ± 0.28	1037.2 ± 1.0	1036.3 ± 1.1
BT-0.05	3.96 ± 0.20	4.16 ± 0.23	3.12 ± 0.03	3.00 ± 0.04	5.16 ± 0.08	5.12 ± 0.06	0.73 ± 0.01	0.70 ± 0.01	10.56 ± 0.28	10.66 ± 0.23	1037.1 ± 0.8	1037.6 ± 0.8
BT-0.1	4.00 ± 0.31	4.14 ± 0.19	3.07 ± 0.05	2.97 ± 0.03	5.23 ± 0.04	5.08 ± 0.10	0.72 ± 0.01	0.69 ± 0.01	10.65 ± 0.21	10.62 ± 0.31	1036.8 ± 1.1	1036.9 ± 1.3
BT-0.25	4.05 ± 0.27	4.15 ± 0.22	3.17 ± 0.03	3.09 ± 0.03	5.17 ± 0.10	5.14 ± 0.06	0.74 ± 0.01	0.72 ± 0.01	10.69 ± 0.20	10.70 ± 0.28	1037.3 ± 0.7	1038.2 ± 0.7
BT-0.5	4.00 ± 0.21	4.10 ± 0.29	3.23 ± 0.04	3.18 ± 0.04	5.18 ± 0.07*c*	5.09 ± 0.10	0.75 ± 0.01	0.74 ± 0.01	10.61 ± 0.28	10.63 ± 0.33	1038.3 ± 1.1	1037.7 ± 1.0
BT-1	3.93 ± 0.18	4.13 ± 0.26	3.17 ± 0.02	3.12 ± 0.03	5.20 ± 0.07	5.11 ± 0.08	0.74 ± 0.01	0.73 ± 0.01	10.49 ± 0.35	10.63 ± 0.28	1038.5 ± 0.7	1037.8 ± 1.1

Data are the mean ± standard deviation of three independent replicates (*N* = 3). Different letters in the same row indicate significant differences (*P* < 0.05).

**Table 5:** Sensory scores and color measurements of Symbilact sample (1-day storage) with added different starches.

Symbilact sample	Sensory 1 day					Color 1 day		
	Appearance	Odor	Texture	Taste	Overall	*L*∗	*a*∗	*b*∗
	(0-1)	(1-3)	(1-10)	(1-10)				
Control	1 ± 0	2.3 ± 0.6	6.7 ± 0.6	4.0 ± 1.0	14.0 ± 1.0	100.00 ± 0.00	−7.98 ± 0.04	25.13 ± 1.24
Native	0 ± 0^a^	1.3 ± 0.6	3.0 ± 1.0^a^	5.7 ± 0.6	10.0 ± 0.0^a^	100.00 ± 0.00	−5.18 ± 0.03	11.43 ± 0.04
АT-0.05	1 ± 0	2.0 ± 0.0	7.0 ± 0.0	6.3 ± 0.6	16.3 ± 0.6	93.50 ± 1.41	−3.28 ± 0.01	7.45 ± 0.01
АT-0.1	1 ± 0	2.0 ± 1.0	8.0 ± 1.0	7.3 ± 0.6	18.3 ± 0.6^b^	99.31 ± 0.42	−3.24 ± 0.01	8.35 ± 0.04
АT-0.25	1 ± 0	2.3 ± 0.6	6.3 ± 1.2	5.7 ± 0.6	15.3 ± 1.2	100.00 ± 0.00	−3.63 ± 0.04	7.69 ± 0.03
АT-0.5	1 ± 0	3.0 ± 0.0	7.3 ± 0.6	8.0 ± 1.0	19.3 ± 1.5^b^	100.00 ± 0.00	−7.05 ± 0.07	16.00 ± 0.03
АT-1	1 ± 0	2.0 ± 0.0	7.7 ± 0.6	8.7 ± 0.6	19.3 ± 1.2^b^	100.00 ± 0.00	−3.90 ± 0.04	9.67 ± 0.03
BT-0.05	1 ± 0	1.7 ± 0.6	6.7 ± 0.6	6.7 ± 0.6	16.0 ± 1.0	100.00 ± 0.00	−4.47 ± 0.14	11.85 ± 0.07
BT-0.1	1 ± 0	1.7 ± 0.6	6.7 ± 0.6	8.0 ± 0.0	17.3 ± 0.6	92.41 ± 0.85	−2.98 ± 0.01	7.29 ± 0.03
BT-0.25	1 ± 0	1.7 ± 0.6	7.3 ± 0.6	6.3 ± 0.6	16.3 ± 0.6	96.42 ± 2.26	−2.95 ± 0.01	9.03 ± 0.03
BT-0.5	1 ± 0	2.7 ± 0.6	7.7 ± 0.6	7.7 ± 0.6	19.0 ± 0.0^b^	100.00 ± 0.00	−4.53 ± 0.03	12.55 ± 0.01
BT-1	1 ± 0	2.7 ± 0.6	8.7 ± 0.6^b^	8.3 ± 0.6	20.7 ± 1.2^b^	100.00 ± 0.00	−5.51 ± 0.03	13.28 ± 0.00

*L*∗: darkness-lightness (0–100); *a*∗: greenness-redness (−60–+60); *b*∗: blueness-yellowness (−60–+60). Data are the mean ± standard deviation of three independent replicates (*N* = 3). Different letters indicate significant differences (*P* < 0.05).

## Data Availability

The data used to support the findings of this study are included within the article. If needed, the data that support the findings of this study are available from the corresponding author (E.V. Nikitina) upon reasonable request.
